# E-mail communication patterns and job burnout

**DOI:** 10.1371/journal.pone.0193966

**Published:** 2018-03-08

**Authors:** Claudia P. Estévez-Mujica, Eric Quintane

**Affiliations:** 1 Department of Psychology, Universidad de los Andes, Bogotá D.C, Colombia; 2 School of Management, Universidad de los Andes, Bogotá D.C, Colombia; Beijing University of Posts and Telecommunications, CHINA

## Abstract

A considerable body of research has documented the negative effects of job burnout on employees and their organizations, emphasizing the importance of the identification of early signs of the phenomenon for the purposes of prevention and intervention. However, such timely identification is difficult due to the time and cost of assessing the burnout levels of all employees in an organization using established scales. In this paper, we propose an innovative way to identify employees at risk of job burnout by analyzing their e-mail communication patterns. Building on the Job Demands–Resources model, we theorize about the relationship between e-mail communication patterns and levels of employee exhaustion and disengagement (two dimensions of burnout). We analyzed 52,190 e-mails exchanged between 57 employees of a medium sized R&D company over a five-month period. We then related these employees’ communication patterns to their levels of burnout, collected using an established scale (the OLBI–Oldenburg Burnout Inventory). Our results provide support for the overall proposition of the paper, that e-mail communications can be used to identify individuals at risk of job burnout. Our models explain up to 34% of the variance of burnout and up to 37% and 19% respectively of the variance of exhaustion and disengagement. They also successfully distinguish between employees with a higher risk of burnout and those with lower levels of risk (F1 score of 84% with recall of 100% and 73% precision). We discuss the implications of our results and present suggestions for future research.

## Introduction

Given the negative effects of burnout on individuals’ wellbeing (e.g., [1–3]) and its economic consequences for employees, organizations and society as a whole [1, 4–6], job burnout has captured the attention of a variety of professionals, including academics, doctors and managers. Problems associated with burnout are estimated to cost more than $120 billion USD a year in the U.S. alone [7]. While burnout was first identified as an issue in the healthcare and customer service industries, it has subsequently been reported to affect employees in a variety of occupations [4, 8, 9] and in multiple geographical and cultural contexts [10–12]. Decades of research have helped identify the causes, manifestations and consequences of burnout (for reviews, see: [4, 11, 13, 14]), and specialized scales such as the Oldenburg Burnout Inventory (OLBI) [8], the Burnout Measure [15]; the Maslach Burnout Inventory (MBI) [16] and the Copenhagen Burnout Inventory [17] have been created to identify employees experiencing burnout. Early detection of burnout is critical to intervention [18]. However, large scale burnout intervention strategies may be difficult to implement as the regular administration of burnout scales via survey to entire organizations is costly and time consuming.

Using the Job Demands–Resources (JD-R) model as a conceptual framework, we propose an innovative method to identify employees at risk of job burnout by monitoring their e-mail communication patterns. We build on the literature on information overload, stress and burnout to propose that communications in general, and e-mail communications in particular, may be conceived either as job demands or as job resources, both of which are related to burnout [19]. More specifically, and without claiming a causal relationship between e-mail communication patterns and employee burnout, we propose that certain patterns of e-mail communication could provide useful information regarding levels of employee job burnout. We relate patterns of e-mail exchange to two well-recognized dimensions of burnout: exhaustion and disengagement, and to a burnout risk index. The key advantage of using e-mail communication patterns as a first step in identifying burnout is that it is an unobtrusive data-collection method [20, 21] free of common variance. It is also cost-effective, allowing continuous monitoring of large numbers of employees [20].

We distinguish between three types of e-mail communication patterns that may be related to the level of job burnout experienced by employees: (i) e-mail volume, (ii) employee position in an e-mail communication network, and (iii) employee e-mail communication behaviors. First, the existing literature indicates that e-mail communication load may be related to job demands or resources for employees [22–24], while jobs demands and resources have been related to the level of burnout that employees experience [8,19]. We therefore examine whether the volume of e-mail communications is related to burnout. Second, the social networks literature has shown that certain positions in communication networks provide employees with access to and control over resources [25]. However, these positions can also be more demanding and may be associated with employee experiences of stress and burnout [26, 27]. Thus, we postulate that the level of burnout employees experience might be related to the position they occupy in their organization’s network of e-mail communications. Third, and finally, the existing literature suggests that some communication behaviors are related to exhaustion [28] and that e-mail misuse or overuse can be qualitatively connected to burnout [29].

To test the validity of e-mail communication patterns as an indicator of employee risk of burnout, we collected burnout and e-mail data from a medium sized R&D company. The burnout data was obtained by administering a standard scale, the OLBI [8], to employees in the company as an online questionnaire. In contrast to previous research that used employee reports or short extracts of communication exchanges to measure e-mail communications, we collected all employee e-mail communications (without collecting any content) during a five–month period leading up to the survey. We also gathered basic demographic and organizational information for each employee. With this information, we estimated two different types of models: linear regression models to explain the variance of burnout and logistic regression models to distinguish between employees at a high and low risk of burnout. In doing so, we respond to calls in the literature for the identification of individuals at high risk of burnout (see: [11]).

Our results provide support for the overall proposition of the paper, which hypothesizes that e-mail communications could be used to identify individuals at risk of burnout. Our models explain up to 34% of the variance in the burnout risk index and 37% and 19% respectively of the variance of exhaustion and disengagement, two of the constituent dimensions of burnout. Our models also successfully distinguish between employees at higher risk of burnout and those at lower risk (F1 score of 84% with a recall of 100%). These results are important, as they open the door to using e-mail communication patterns as an early warning system–a first step–in identifying employees at risk of burnout. While additional research is needed to replicate our results in different contexts, and to improve the predictive power of our indicators, e-mail communication patterns may prove to be a cost effective and efficient way to continuously assess the risk of employee job burnout in organizations, thus improving intervention strategies designed to prevent its negative consequences.

### Theory

#### Burnout

The most widely accepted definition of burnout has been proposed by Maslach, Schaufeli and Leiter [30], who define the syndrome as a “prolonged response to chronic emotional and interpersonal stressors of the job” (p.1). While burnout was originally identified among health care professionals, nowadays academics recognize its occurrence in a wide variety of work environments [8, 9]. Experiencing burnout is associated with at least two consequences for employees. First, it is related to decreased health. The extant literature has documented the co–occurrence of burnout and a variety of psychological and physical health problems including depression [1, 31] anxiety [32], and physical illness [33]. Second–and principally because of its effects on employee well–being and health–burnout is related to decreasing work-related outcomes, such as reduced performance [34], lower levels of engagement and absenteeism [35].

#### Job Demands-Resources model

The JD-R model is one of the most widely-used models for studying job burnout [19]. The core premise behind the model is that job characteristics can be classified as either “job demands” or “job resources” [36]. Job demands refer to characteristics of work that produce strain and are therefore related to costs for the employee [37]. Conversely, job resources refer to aspects of work that allow employees to accomplish tasks, which in turn relate to individual development [37]. Hence, job resources buffer the impact of job demands and are associated with benefits that accrue to the individual [37].

The JD-R model relates job demands and resources to two different dimensions of burnout: exhaustion and disengagement. Exhaustion, predicted by job demands (see: [19, 37]), refers to an after-effect of “intense physical, affective or cognitive strain” [8] (p. 328). Disengagement, predicted by job resources (see: [19, 37]), is related to standing aside and having negative attitudes towards work [8]. By contrast, worker engagement has the opposite effect on burnout [18]: engaged individuals are positive about their work and feel involved in it [18]. Hence, in the JD-R model, demanding aspects of work (e.g., physical workload, time pressure, recipient contact, physical environment, and shift work) lead to exhaustion [36], while a lack of job resources (e.g., feedback, rewards, job control, participation, job security, and supervisor support), lead to disengagement [36]. Finally, an imbalance between the two (i.e., when job demands exceed job resources) leads to burnout in which employees exhibit both exhaustion and disengagement [19, 36, 37].

#### Communications, e-mailing and burnout

Researchers have long investigated the relationship between communications, stress and burnout. Starting in the 1980s, communication researchers became interested in the impact of the quantity and quality of work communications on employee wellbeing, stress and burnout. Johnson and Indvik [38] conceptualized the relationship between the quantity and quality of certain communication behaviors and stress/burnout, while Albrecht, Irey and Mundy [26] and Ray [27] analyzed how positions in a communication network might be related to experience of stress and burnout. Subsequently, with the advent of new communication technologies, researchers examined how the use of some communications tools was related to the experience of strain (e.g., [39, 40]). Overall, this stream of research found that different aspects of work communications and particular individual network roles are related to increased demands and loss of control, which, in turn, are associated with stress, exhaustion and job burnout. As such, this research has provided support to arguments claiming a relationship between work communications and employee experiences of stress and burnout.

The increase of e-mail communication as a tool for information sharing [41] has led researchers to examine the relationship between e-mail communications, stress and burnout in more detail. These studies generally suggest that excessive use of e-mail might be an antecedent for employee burnout. For example, Camargo’s qualitative analysis [29] shows that e-mail overuse can cause information overload, while the stress related to continuously answering e-mails may be an antecedent of burnout. Building on the JD-R model, Derks and Bakker [23] provide a theoretical account of the impact of e-mail communication on work. They suggest that even though e-mails might be seen as a resource and a tool used by employees to engage with their workplace, excessive e-mail volume can turn into a costly job demand.

Further empirical studies have confirmed the relationship between excessive e-mailing and exhaustion. Barley, Meyerson & Grodal [42] used e-mail logs of employee communications over a period of two working days, to show that the time spent on writing or answering e-mails was positively related to feelings of overload measured by the exhaustion dimension of the MBI, while interviews with employees confirmed the association between communication–related feelings of stress and volume of e-mails. Brown et al. [43] used surveys to examine the role of e-mailing as a workplace stressor and its association with emotional exhaustion. Using employees’ estimations of how many e-mails they exchanged daily as well as a one-day monitoring of employees’ actual communications, they found that employees who handle higher volumes of e-mails are more likely to report feelings of e-mail overload. This, in turn, mediated a positive relation between e-mail volume and emotional exhaustion. These studies suggest that an overload of e-mail communications can be related to higher reports of stress and exhaustion. However, answering e-mails may also act as a coping strategy to deal with overload.

In sum, there is evidence that work communications are related to stress and burnout and that, in particular, e-mail communications seem to be related to the exhaustion dimension of burnout. However, the empirical evidence provided by the studies that relate e-mail communications to exhaustion and burnout is still limited, either because they use self–reports of e-mail communication patterns by individuals or because their e-mail usage observation periods are very short. On the other hand, no study, to our knowledge has related e-mail communications to the disengagement dimension of burnout. Our aim is to provide stronger empirical evidence of the relationship between e-mail communication patterns and burnout by systematically relating patterns of e-mail exchanges over an extended period of time to the level of exhaustion, disengagement and burnout experienced by employees.

In the following section, we theorize about how different types of e-mail communication patterns could be related to exhaustion, disengagement and, more generally, to job burnout. In order to do so, we build on the JD-R model and consider the argument that e-mail communications can act as job demands and resources for employees.

### Hypotheses

Identifying individuals at risk of burnout is a necessary first step in preventing its development. Building on prior research, and using the JD-R model as a reference, we develop a series of hypotheses relating individual e-mail communication patterns to job exhaustion, disengagement and risk of burnout. We distinguish between three types of e-mail communication patterns: (i) patterns based on the volume of e-mail communications, (ii) patterns associated with the position of employees in an e-mail communication network, and (iii) patterns related to employees’ specific e-mail communication behaviors.

#### Burnout and e-mail volume

We argue that receiving a higher volume of e-mails is related to increased exhaustion and burnout. The JD-R model states that individuals who experience higher levels of demands in their jobs might experience increased levels of exhaustion, which might in turn lead to burnout [19, 37]. The literature has recognized employee workload as a source of job demand [4]. One way to quantify workload is through the number of requests that employees receive to perform tasks. Previous research on e-mail use has pointed out that e-mail communications are closely related to job tasks [29, 41, 44]. In fact, it is also well recognized that e-mail is not only a means of communication but also a task management system through which tasks are distributed (see: [24, 29]). Consequently, the number of e-mails received by employees can be an indicator of the extent to which colleagues request them to accomplish certain tasks, thus providing information about the workload of individual employees. Following this argument and in line with previous research suggesting that e-mail volumes are related to increases in the number of hours worked [42], perceptions of e-mail overload [43, 45] and emotional exhaustion [43], we hypothesize that higher volumes of e-mails received could indicate increased levels of job demands, which could in turn be related to increased exhaustion and a greater risk of developing burnout.

Hypothesis 1: The higher the volume of e-mails an employee receives, the higher his or her levels of exhaustion and the greater the risk that the employee will develop job burnout.

We argue that the higher the volume of e-mails an employee sends, the lower his or her level of disengagement and burnout. An alternative way to consider e-mail communication is as a tool through which employees can actively engage with their work. Employees send e-mails to communicate with colleagues, to answer requests, to share information and to manage tasks. That is, it is a job resource that allows individuals to control their work demands. In this regard, Barley, Meyerson & Grodal [42] found that the higher the volume of messages employees handle, the more they feel they can cope with their workload. Based on this and on the association between job resources and disengagement theorized in the JD-R model [19, 37], we propose that a higher number of e-mails sent could represent attempts by employees to engage with their work (i.e., an absence of disengagement). Thus, sending large numbers of e-mails could operate as a job resource, indicating lower levels of disengagement and a lower risk of developing burnout.

Hypothesis 2: The higher the volume of e-mails an employee sends, the lower his or her levels of disengagement, and the lower the risk that the employee will develop job burnout.

#### Burnout and the social position of employees in an e-mail communication network

An alternative way to relate e-mail communication patterns to the risk of developing burnout is by examining the position of employees in an e-mail communication network (e.g., [26, 27]). There is considerable empirical research associating an individual’s position in communication or advice networks with specific outcomes for the individual (see: [46]). Many of the positive outcomes of occupying a central position in a social setting are related to access to and control over information (e.g., [25, 47]). Attention has been paid to the consequences of occupying a central position in a communication network [46], including an e-mail communication network [48]. The more central individuals are in a communication network, the better able they are to obtain and accumulate knowledge and information that can be used as a resource to solve problems and to exchange information with coworkers [48]. More central individuals are also more satisfied with their work, while employees in peripheral positions tend to have lower levels of satisfaction [49, 50]. Thus, we propose that a central position in an e-mail communication network enables employees to access resources that would otherwise not be available, affecting employees’ engagement with their work. By contrast, a peripheral position reduces the amount of resources employees are able to access, in turn affecting their job satisfaction and engagement.

Hypothesis 3a: The more central the position of an employee in an e-mail communication network, the lower his or her levels of disengagement and the lower the risk that the employee will develop job burnout.

Social networks literature has demonstrated that it is not only the centrality of individuals in a social network that provides them with access and control over information and resources, but the extent to which their alters (i.e., the individuals with whom they communicate) are connected [51]. Individuals occupying brokerage positions–that is, whose alters are not connected–are argued to have earlier access to more diverse information, as well as exerting more control over it [25]. It has been demonstrated that this also leads to advantageous outcomes for the individuals occupying such positions [51, 52]. Since increased access to resources is related to higher engagement and to a lower risk of developing burnout [18, 19, 37], we propose that:

Hypothesis 3b: The less connected the alters of an employee in an e-mail communication network, the lower the levels of disengagement and the lower the risk that the employee will develop job burnout.

However, the centrality of an employee in an e-mail communication network may also be associated with stress, caused by receiving demands from many individuals. We argued that while a central position increases the amount of resources available to the employee occupying it, it might also imply that the employee is in contact with many alters and hence potentially subject to increased demands from these alters. As such, a central position in an e-mail communication network, inasmuch as it indicates an increased number of communication partners and potential requests from these partners, may generate a loss of control over the demands made on the employee [23], in turn, possibly increasing the risk of developing burnout.

Hypothesis 4a: The more central the position of an employee in an e-mail communication network, the higher his or her levels of exhaustion, and the higher the risk that the employee will develop job burnout.

We also argue that individuals whose alters are not mutually connected are, alternatively, likely to experience higher levels of exhaustion and burnout. Early research on communication networks found that “linkers” (i.e., individuals who link heterogeneous people) experience higher levels of burnout than individuals occupying other positions, as measured by an earlier burnout measure: the Tedium Scale [26, 27]. This is because employees who intermediate communication flows between disconnected alters will have to act as interfaces between different individuals [25], increasing the demands that are placed upon them, thereby affecting the levels of exhaustion they experience and their risk of suffering job burnout.

Hypothesis 4b: The less connected the alters of an employee in an e-mail communication network, the higher the levels of exhaustion and the higher the risk that the employee will develop burnout.

#### Burnout and communication behavior

Burnout risk may also be associated to specific e-mail communication behaviors rather than to e-mail volume or an employee’s social position in the network. We explore three specific communication behaviors that might be related to differences in the risk of burnout: (i) the extent to which employees are in control of the flow of e-mails in their mailboxes (indicated by the ratio of e-mails sent versus e-mails received); (ii) the extent to which employees engage in sending work e-mails outside office hours (at night, during holidays and weekends); and (iii) the number of hierarchical superiors with whom employees have frequent, reciprocated communications.

First, a loss of control of the flow of e-mails might occur when the volume of information employees need to process exceeds their capacity to do so [53]. Hence, employees must attain a balance between their information load and their processing capabilities. If this balance is absent, they might experience information overload [44, 54], which may lead to stress [55] and burnout-related exhaustion [42, 43]. Following the same logic, it can be argued that overload occurs when the volume of demands exceeds an employee’s capacity/resources to deal with all the demands. We propose that one way to capture the occurrence of overload is by measuring the ratio of the number of e-mails sent (resources), and the number of e-mails received (demands). In this case, according to the JD-R model, an imbalance between e-mails sent and e-mails received can be perceived as a loss of control, which, in turn, can be related to burnout. Hence, we propose that lower values for the ratio between e-mails sent and received–indicating that employees receive more e-mails than they send–might be a sign of having more demands than resources.

Similarly, sending e-mails but not receiving a comparable number of responses might also occur in situations in which employees attempt to engage with the workplace, but their engagement is not reciprocated. Research on fairness and burnout has pointed to the fact that imbalanced social exchanges can be predictive of burnout (see: [18]). Consequently, based on the logic that an asymmetry between the number of e-mails sent and the number of e-mails received represents an imbalance between job demands and job resources, we propose a curvilinear relationship between the ratio of e-mails sent and received and the risk of developing burnout.

Hypothesis 5: The ratio of e-mails sent versus e-mails received will have a curvilinear relation to the risk of burnout experienced by employees, such that lower and higher values for the ratio will be associated with a higher risk that the employee will develop burnout.

Second, an object of extensive research in the communication literature is the invasion of non–working times and places by work-related communication. This stream of research argues that even though communication-related technologies are flexible and convenient; this flexibility can negatively affect employee wellbeing (see: [28]). Communications that occur outside regular working hours spill into employees’ lives [23]. Barley, Meyerson & Grodal [42] found that employees extend their working days by e-mailing early in the morning, at night and during weekends, as a strategy to handle their backlog of e-mails, which would otherwise act as a constant reminder of how overloaded they are. Moreover, researchers have documented evidence that communications outside working hours are related to psychological distress, sleep problems [56] and increases in stress [55]. Similarly, Wright et al. [28] found that employee reports of engaging in communication activities outside working hours are related to perceptions of work intrusions into personal life, which can predict the exhaustion dimension of job burnout. Accordingly, we propose that the number of e-mails sent at night and during holidays and weekends might represent employees’ need to have to work outside working hours in order to complete the tasks demanded of them. We propose that this is related to exhaustion and to the risk of developing burnout. Thus:

Hypothesis 6a: The more an employee sends e-mails during out-of-office hours (nights, holidays and weekends), the higher his or her levels of exhaustion and the higher the risk that the employee will develop burnout.

However, we could argue the possibility of a contrasting relationship between e-mails sent during out-of-office hours and exhaustion and burnout. The extant literature has shown that experiencing exhaustion and burnout occurs when employees feel that they cannot control their workload [42]. Handling e-mailing during out-of-office hours can indicate efforts made by employees to manage their workload and avoid losing control [42]. As such, this pattern of e-mailing might also be related to increased levels of control over work, and thus to higher employee engagement, which may, in turn, be related to lower risks of developing burnout.

Hypothesis 6b: The more an employee sends e-mails during out-of-office hours (nights, holidays and weekends), the lower his or her levels of disengagement and the lower the risk that the employee will develop burnout.

Another way in which specific patterns of e-mail communications can be related to the risk of burnout is as a reflection of employees’ communication behaviors towards their superiors. Superiors are typically a key source of job demands, which the employee has limited leeway to refuse, delay or otherwise manage. Reciprocation is important, as it signals a process by which the employee and the superior are working in conjunction to complete a task [21]. We argue that a continuous back and forth in communications between employees and their hierarchical superiors indicates an increased level of oversight and of demands imposed by the latter. Hence, the higher the number of hierarchical superiors with whom an employee communicates in a reciprocal manner, the higher the demands that are placed on the employee, and the less discretion he or she has in managing these demands. We therefore propose that individuals with a higher number of hierarchical superiors with whom they have reciprocal communications will experience higher levels of exhaustion and be at greater risk of developing burnout.

Hypothesis 7: The higher the number of reciprocated e-mail communication ties an employee has with hierarchical superiors, the higher his or her level of exhaustion and the greater the risk that the employee will develop burnout.

## Materials and methods

### Data collection

We collected data on e-mail use, burnout, basic demographics and the hierarchical level of employees in a medium–sized R&D company in Italy. The research project was approved of by the Ethics Committee of the School of Management at Universidad de los Andes. Employees were briefed about the project during a presentation. Key information about the project was provided intelligibly on the first page of the electronic survey and respondents provided informed consent electronically before continuing with the survey. The online survey provided an explanation of the study, including its purpose and the fact that all answers would be treated confidentially. Respondents were very clearly informed that they were not obliged to complete the survey and they could stop responding at any point. Data collection took place between January 7^th^ and May 25^th^ 2015, when the company had a total of 212 employees distributed across five hierarchical levels (general manager, executives, managers, staff members and operational workers). We collected burnout data via an online survey on the 25th May 2015, using the OLBI questionnaire. We also collected demographic information for every employee (i.e., age, gender), as well as their hierarchical level. All the data collected was de-identified before analysis, using unique code numbers for each employee.

All internal company e-mail data (communications between company employees) was collected over a five–month period between January and May 2015. The e-mail data included information about the sender, the recipient and the time (date and hour) of the e-mail but not about its content. Since we were interested in e-mails as a proxy of employees’ actual resources or demands, we removed mass distribution e-mails (i.e., e-mails sent to more than 4 individuals) from the analysis, according to an approach and a threshold that were consistent with previous literature using e-mail data [20, 57, 58]. Our e-mail dataset records include 67,651 e-mail exchanges between 160 employees (the remaining employees did not send or receive any e-mails during the period of analysis).

### Sample

Because we were interested in explaining employee e-mail communication patterns as they relate to burnout, we needed to ensure that the employees included in the study used e-mail as part of their daily work. A portion of the employees of the R&D company produce prototypes of the products it designs, and work on the manufacturing line without the need to regularly interact via e-mail for work purposes. These employees were removed from the sample, as were those who sent fewer than 10 e-mails over the five–month period (i.e., fewer than two per month). We also removed one employee who sent more than 3,700 e-mails (approx. 11 SD more than the average for our sample). Thus, the total number of employees included in our final dataset with both complete e-mail records and burnout information was 57.

Our final sample of 57 employees was made up of three different hierarchical levels (26 staff members, 28 managers and 3 executives), with ages ranging from 26 to 55 (x = 41.50), and 79% of whom were men. These 57 employees exchanged a total of 52,190 e-mails over 20 weeks. On average, the total number of e-mails exchanged between these employees on every complete week was 2,645 (SD: 256.72). The weekly average number of e-mails sent per employee was 25.05 (SD: 17.16) and the weekly average number of e-mails received was 36.11 (SD: 26.10). The maximum number of emails sent on a given week by a single employee was 139 e-mails and the maximum number of emails received by a single employee during a week was 198.

### Variables and controls

#### Dependent variables

Burnout data was collected using an online version of the OLBI [8] questionnaire, measuring burnout using positively and negatively framed items according to two separate subscales: exhaustion and disengagement (the same dimensions of burnout used in the JD-R model). The exhaustion dimension has eight items related to tolerance to pressure, ability to manage workload and feelings of tiredness (e.g., “After my work, I usually feel worn out and weary”, “I can tolerate the pressure of my work very well”, “Usually, I can manage the amount of work I have very well”). The disengagement dimension has eight items related to current job engagement, enjoyment and satisfaction (e.g., “I always find new and interesting aspects in my work”, “Sometimes I feel sickened by the tasks I have to perform at work”, “Lately, I tend to think less at work and do my job almost mechanically”). We translated the original items into Italian and then had them back–translated into English by an independent translator to ensure language equivalence. Participants were instructed to use a 7–point Likert-type scale to assess how much they agreed with each statement (7 = Totally agree, 1 = Totally disagree).

Positively–framed items were reverse–coded to assess burnout. Factor analysis, using principal axis factoring and oblimin rotation, points to moderate/adequate fit of items for a structure with two dimensions (KMO = .86, Bartlett´s test: chi-square (78) = 377.93 p< .01). Due to poor factor loadings, we removed item 13 of the disengagement dimension from the analysis (“This is the only type of work I can imagine myself doing”). In addition, because of incorrect and poor factor loadings, and in order to make our burnout assessment as similar as possible to the original OLBI questionnaire, we removed items 5 and 7. All factor weights were greater than 0.3 (See Additional analysis section for a robustness check of our results using a 0.7 cut-off point). There was moderate-to-strong correlation between scales (.56 p < .001). Means, ranges, standard deviations, Cronbach’s alphas and correlations for the exhaustion and disengagement dimensions of burnout are presented in Table 1. Exhaustion was computed as the average of the exhaustion items and had values ranging from 1 to 5.57. Disengagement was calculated as the average of the disengagement items and had values ranging from 1 to 5.3. A burnout index was computed as the average of exhaustion and disengagement, with values ranging from 1.14 to 5.15 (x = 3.06).

**Table 1:** Means, ranges, standard deviations, Cronbach’s alphas, and correlations for each dimension of the burnout scale (exhaustion and disengagement).

	Mean	Min.	Max.	SD	1	2
Exhaustion	3.20	1	5.57	1.07	(.85)	
Disengagement	2.90	1	5.33	.99	.56***	(.86)

Cronbach’s alphas are reported in the diagonals for each respective subscale. Dimensions were rated on 1–7 scale. The exhaustion dimension comprises 7 items and the disengagement dimension comprises 6 items.

***p < .01.

We defined individuals at risk of developing burnout as belonging to one or both of the following two categories. First, following Maslach and Leiter [18], we considered that individuals who report medium to high scores on the exhaustion or disengagement dimensions of burnout may be considered to be at risk of developing burnout. Second, we considered that individuals whose average score across the exhaustion and disengagement dimensions (burnout index) ranged from medium to high are also at risk of developing burnout. Employees may be experiencing a high level in one dimension and a low level in the other, or medium levels in both, indicating potential burnout. We are aware of criticisms leveled against aggregating burnout dimensions [59]. However, these studies sought to identify individuals who had already developed full burnout syndrome. Since our goal is to identify individuals who were *at risk* of developing burnout, a compound burnout index based on the exhaustion and disengagement dimensions is a useful and valid early indicator of the likelihood of employees developing job burnout.

#### Independent variables

Using the e-mail communication data that we collected, we created a matrix that lists employees in rows and columns. Each cell of the matrix contains the number of e-mails sent from the employee in the row to the employee in the column. This matrix represents the e-mail communication network of our sample. To represent more stable communication exchanges, we also computed a “strong” e-mail communication network in which a cell contains a value only if the amount of e-mails sent from employee *i* to employee *j* is higher than the average number of e-mails sent by all the other possible pairs of employees (i.e., 16 e-mails; cases of zero e-mails exchanged excluded).

We computed volume, positional and behavioral variables for all individuals in our sample, including e-mail exchanges with other employees in the organization even if they were not included in our sample. The rationale for this was that e-mails exchanged with any other company employee, even if not included in the sample, might represent actual resources or demands. All variables were standardized before analysis.

*Volume Variables*. Volume variables refer to the number of e-mails sent and received by each employee. To be as precise as possible in our representation of e-mail volumes, we computed multiple versions of e-mails sent and e-mails received: (i) the sum of all e-mails sent or received, (ii) the average daily number of e-mails sent or received over the period of analysis (cases of zero e-mails exchanged excluded), and (iii) the total volume of e-mails sent or received, compared to a hierarchical level baseline (calculated as the sum of all e-mails sent or received by an employee minus the average volume of e-mails sent or received by all other employees at the same hierarchical level).

Preliminary analyses using each of these variables as possible predictors of the presence and dimensions of burnout show that: (i) for *E-mails Received*, the indicator that provided the best prediction of burnout and of its dimensions was the daily average number of e-mails received by an employee during the period of analysis and (ii) for *E-mails Sent*, the indicator that provided the best prediction of burnout and its dimensions was the total number of e-mails sent by an employee compared to his/her hierarchical level baseline.

Further, we used the method proposed by Xuan and Filkov [60] to estimate the number of tasks that employees have to undertake by calculating the number of bursts of e-mails that they experience in a given amount of time. A burst is defined as a sequence of e-mails that occurs within a specific time threshold between each e-mail. We calculated ten burst variables corresponding to the number of bursts for each employee for periods of time ranging from 1 to 10 days. Preliminary analyses showed that the use of burst variables did not increase the variance explained for our linear regression models and burst variables were not significant. By contrast, the one-day burst variable slightly improved recall and F1 statistics for burnout and exhaustion in our penalized logistic regression models.

*Positional Variables*. Positional variables represent the position of each employee in the structure of the network of e-mail communications in the organization. Positional variables were calculated using the organizational communications network using all company e-mails exchanged during the period of analysis. We computed *Degree–*the number of communication partners each employee has–using UCINET’s ego network unweighted basic measures procedure [61]. We computed *Constraint–*the extent to which the alters of each employee were connected to each other [25]–using UCINET´s ego networks structural holes procedure [61]. Employees with high values of constraint have many alters who communicate with each other, while employees with low values of constraint have many communication partners who do not communicate with each other. Following Burt [25, 51], we replaced values of 0 and values above 1 with 1 and calculated the natural logarithm of the value.

*Behavioral Variables*. Behavioral variables represent specific patterns of employee communication behaviors. We calculated three different indicators of communication behaviors: *Ratio Sent/Received*, *E-mails Sent During Out-of-office Hours* and *Higher Hierarchical Level Reciprocity*.

To measure the balance (or lack thereof) between sent and received e-mails, we computed a *Ratio Sent/Received* parity indicator. This indicator is the total number of e-mails sent by an employee divided by the total number of e-mails received by this same employee over the observation period. We computed the squared value of the *Ratio Sent/Received* in order to capture the curvilinear effect in which we were interested.

To measure the number of e-mails employees sent during out-of-office hours, we computed two preliminary indicators: (i) the sum of the number of e-mails sent at night and during holidays and weekends over the observation period and (ii) the daily average of the number of e-mails sent at night and during holidays and weekends over the same period. Preliminary analyses using each of these variables as possible predictors of burnout and its dimensions show that the daily average of e-mails sent during out-of-office hours provides the best prediction for burnout and its dimensions. This variable was computed as the average of the daily number of e-mails sent (cases of zero e-mails exchanged excluded) at night (6:00pm to 8:00am, during which lapse of time the company’s e-mail communications first decline and then increase again), holidays (2 days during the observation period, according to the Italian calendar) and weekends (Saturdays and Sundays, 40 days).

We calculated the number of reciprocal communication ties an employee has with employees at higher hierarchical levels (*Higher Hierarchical Level Reciprocity*) by counting the number of employees at a higher hierarchical level with whom a given employee has strong reciprocal ties. We only used strong ties in the e-mail communication network because the network that included all e-mail communications was almost entirely reciprocal and because our theory requires evidence of the occurrence of more frequent demands placed on the employee from above.

#### Control variables

We included the age (standardized), the gender (1 = female) and the hierarchical level (categorical) of each employee in our models. While previous studies have not proposed a definitive pattern for the relation between age and burnout, some evidence exists for a possible negative correlation between the two [30, 62]. Similarly, even though the results from previous studies are ambiguous, they support the idea that gender is an important variable that helps to explain burnout [30]. Also, aware of possible differences between hierarchical levels, we included two dummy variables representing the position occupied by each employee in the hierarchy: *Executive Hierarchical Level* takes the value of 1 if the individual is an executive and 0 otherwise, *Staff Member Hierarchical Level* takes the value of 1 if the individual is a staff member and 0 otherwise.

Descriptive statistics and correlations between all variables are presented in Table 2. Almost all correlations between variables were low to moderate. Exceptions to this pattern occurred between burnout and each of its dimensions and between e-mails sent and received. Given that e-mails first sent by an employee tended to be answered and that e-mails that an employee received tended to be reciprocated, the latter is a normal pattern of e-mail exchange (especially when excluding massive e-mails as in here) [20]. Even though correlations between some of the predictors were moderate, multicollinearity problems for all the models reported below were acceptable (VIF < 5). To avoid multicollinearity problems for the models with volume variables (*E-mails Sent* and *E-mails Received*) we estimated a separate model for each.

**Table 2:** Basic descriptive statistics and Pearson correlation coefficients of variables included in the models (n = 57).

*Variable*	Mean	S.D.	1	2	3	4	5	6	7	8	9	10	11	12	13	14
*1*. *Burnout*	3.06	.93														
*2*. *Exhaustion*	3.20	1.06	.91**													
*3*. *Disengagement*	2.92	.99	.90**	.63**												
*4*. *Gender*	79% men	---	-.06	-.04	-.07											
*5*. *Age*	41.5	8.64	.36**	.33*	.32*	.05										
*6*. *Executive Hierarchical Level*	---	---	-.14	-.14	-.12	-.12	.11									
*7*. *Staff Member Hierarchical Level*	---	---	-.25	-.21	-.24	-.04	-.47**	-.22								
*8*. *E-mails Received*	.00	1.00	-.03	.07	-.14	.31*	.14	.32*	-.32*							
*9*. *E-mails Sent*	.00	1.00	-.18	-.08	-.25	.35**	-.02	.00	-.00	.79**						
*10*. *Degree*	.00	1.00	-.20	-.17	-.19	.46**	-.20	-.08	-.02	.46**	.58**					
*11*. *Constraint*	.00	1.00	-.17	-.19	-.12	-.18	-.09	-.03	.48**	-.26*	-.18	-.36**				
*12*. *E-mails Sent During Out-of-office Hours*.	.00	1.00	-.40**	-.33*	-.40**	-.04	.00	.47**	-.10	.52**	-.54**	.13	-.02			
*13*. *Higher Hierarchical Level Reciprocity*	.00	1.00	.07	.18	-.07	.23	-.24	-.14	.44**	.27*	.52**	.38**	-.13	.12		
*14*. *Ratio Sent / Received*	.00	1.00	-.08	-.09	-.06	.07	-.04	.00	.18	-.16	.27*	.01	-.03	.21	.25	
*15*. *Ratio Sent / Received Squared*	.00	1.00	-.01	-.05	.04	.04	.16	-.11	.29*	-.17	-.06	-.31*	.37**	.07	.10	.47**

**p < .01

*p < .05

## Results

Below, we present the results from two sets of analyses. First, we estimate linear regression models to explain the variance of job burnout and both of its separate OLBI dimensions (exhaustion and disengagement). Second, we use logistic regressions to examine the ability of our models to identify employees with a higher risk of experiencing burnout using exhaustion, disengagement and the burnout index.

### Linear regressions–explaining the variance of burnout

We estimated multivariate linear regression models in order to explain the variance of the burnout index and its dimensions using e-mail data. We present different models for the burnout index and for each of its dimensions, starting with exhaustion, then disengagement and finally the burnout index. All models included control variables (i.e., age, gender and hierarchical level). Predictor variables were included in the models using a stepwise procedure.

Table 3 presents linear regression models predicting the exhaustion dimension of burnout. Model 1 corresponds to the baseline model with all control variables predicting exhaustion. *Age* is positively and significantly related to exhaustion: the older the employee, the higher the levels of OLBI exhaustion (ß = .32, p< .05). This is contrary to what might have been predicted from the existing literature, in which age is typically either unrelated or negatively related to exhaustion [62]. Neither *Gender* nor *Hierarchical level* is significantly related to exhaustion (p>.1). In Model 2, we tested Hypothesis H1 by including the *E-mails Received* indicator. Results show that the volume of e-mails received is not significantly related to exhaustion (p>.1). In Model 3, we tested Hypotheses H4a and H4b by including the positional variables *Degree* and *Constraint*. However, these are only marginally significantly related to exhaustion (ß_Degree_ = -.22, ß_Constraint_ = -.26, p< .1). In Model 4, we included the behavioral variables (*E-mails Sent During Out*-of-*office Hours* and *Higher Hierarchical Level Reciprocity*). Contrary to Hypothesis H6a, we found that the more e-mails an employee sends during out-of-office hours, the lower his or her level of exhaustion (ß = -.45 p< .01). Consistent with Hypothesis H7, the higher the number of strong reciprocal ties an employee has with other employees at higher hierarchical levels, the higher the level of exhaustion (ß = .51. p< .01). In Model 5, we included all variables hypothesized to be related to exhaustion. The final model explains 37% of the variance of exhaustion. In this final model, *Age* and *Constraint* become non-significant (p>.1). Contrary to our hypothesis, *Degree* becomes negative and significant (ß = -.38 p< .01). The more e-mail communication contacts an employee has, the lower his/her level of exhaustion. Consistent with results in Model 4, *E-mails Sent During Out*-of-*office Hours* (ß = -.51 p< .01) and *Higher Hierarchical Level Reciprocity* (ß = .50 p< .05) are unchanged in the final model.

**Table 3:** Linear stepwise regression models–predicting exhaustion (n = 57).

	Model 1	Model 2	Model 3	Model 4	Model 5
Variables	ß (S.E.)	ß (S.E.)	ß (S.E.)	ß (S.E.)	ß (S.E.)
Constant	3.40*** (.22)	3.40*** (.22)	3.25*** (.24)	3.6*** (.19)	3.54*** (.21)
**Step 1: Control Variables**					
Age	.32** (.13)	.32** (.14)	.30** (.14)	.30** (.13)	.21 (.13)
Gender	-.14 (.38)	-.24 (.40)	.01 (.40)	-.44 (.30)	-.30 (.31)
Executive Hierarchical Level	-.97 (.96)	-1.13* (.11)	-.94* (.97)	-.01 (.86)	-.31 (.93)
Staff Member hierarchical level	-.24 (.31)	-.19 (.32)	-.01 (.39)	-.70** (.29)	-.56 (.40)
**Step 2: Volume Variables**					
E-mails Received		.11 (.20)			.30 (.21)
**Step 3: Position Variables**					
Degree			-.22* (.15)		-.38*** (.16)
Constraint			-.26* (.15)		-.08 (.14)
**Step 4: Behavior Variables**					
E-mails Sent During Out-of-office Hours				-.45*** (.12)	-.51*** (.14)
Higher Hierarchical Level Reciprocity				.51*** (.14)	.50** (.18)
R^2^	.15	.16	.21	.39	.47
Adjusted R^2^	.09	.08	.11	.32	.37
ΔR^2^		.01	.06	.24	.32
F	2.37*	1.98*	2.16*	5.42***	4.69***

The table presents linear regressions models predicting the variance of exhaustion. Model 1 is the base model with controls. Models 2–4 include controls and each predictor in a stepwise procedure. Model 5 is the final model including all variables.

Standard errors are robust. Two-tailed tests for all variables.

*p < .1

**p < .05

***p < .01

Table 4 presents linear regression models predicting the disengagement dimension of burnout. Model 1 is the baseline model with all controls predicting disengagement. Consistent with our previous exhaustion results, *Age* is positively and significantly related to disengagement. The older the employee, the higher the levels of OLBI disengagement (ß = .26, p< .05). Neither *Gender* nor *Hierarchical level* is significantly related to disengagement (p>.1). In Model 2, we tested Hypothesis H2 by including the *E-mails sent* indicator. Results show that the volume of e-mails sent is not significantly related to disengagement (p>.1). Similarly, in Model 3, we tested hypotheses H3a and H3b by including the positional variables *Degree* and *Constraint*. However, these variables are not significantly related to disengagement (p>.1). In Model 4, we included the behavioral variable hypothesized to be related to disengagement (*E-mails Sent During Out*-of-*office Hours*). Confirming Hypothesis H6b, the more e-mails sent during out-of-office hours, the lower the level of disengagement (ß = -.43 p< .01). Finally, in Model 5, we included all variables hypothesized to be related to disengagement. The final model explains 19% of the variance of disengagement. In this final model, *Age* becomes non–significant. Consistent with Model 4, *E-mails Sent During Out*-of-*office Hours* (ß = -.47 p< .05) remains significant and negative in the final model.

**Table 4:** Linear stepwise regression models–predicting disengagement (n = 57).

	Model 1	Model 2	Model 3	Model 4	Model 5
Variables	ß (S.E.)	ß (S.E.)	ß (S.E.)	ß (S.E.)	ß (S.E.)
Constant	3.16*** (.22)	3.11*** (.22)	3.08*** (.25)	3.12*** (.20)	3.08*** (.22)
**Step 1: Control Variables**					
Age	.26** (.11)	.26** (.12)	.23* (.13)	.23** (.11)	.20 (.13)
Gender	-.21 (.22)	-.24 (.40)	-.03 (.28)	-.19 (.23)	-.15 (.32)
Executive Hierarchical Level	-.84 (.61)	-.79* (.46)	-.83 (.59)	-.07 (.47)	.14 (.66)
Staff Member Hierarchical Level	-.32 (.29)	-.31 (.30)	-.23 (.41)	-.34 (.27)	-.29(.36)
**Step 2: Volume Variables**					
E-mails Sent		-.24 (.16)			.11(.26)
**Step 3: Position Variables**					
Degree			-.20 (.17)		-.16 (.18)
Constraint			-.13 (.18)		-.09 (.16)
**Step 4: Behavior Variables**					
E-mails Sent During Out-of-office Hours				-.43*** (.13)	-.47** (.24)
R^2^	.15	.20	.18	.29	.31
Adjusted R^2^	.08	.12	.08	.22	.19
ΔR^2^		.05	.03	.14	.16
F	2.28*	2.55**	1.82	4.16***	2.63**

The table presents linear regressions models predicting the variance of disengagement. Model 1 is the base model with controls. Models 2–4 include controls and each predictor in a stepwise procedure. Model 5 is the final model including all variables.

Standard errors are robust. Two-tailed tests for all variables.

*p < .1

**p < .05

***p < .01

Table 5 presents linear regressions models predicting the burnout index. Model 1 is the baseline model with all controls predicting the burnout index. Consistent with previous results, *Age* is positively and significantly related to the burnout index. The older the employee, the higher the levels of the burnout index (ß = .29, p< .05). Neither *Gender* nor *Hierarchical level* is significantly related to burnout index (p>.1). In Models 2 and 3, we tested hypotheses H1 and H2 as they related to the risk of burnout by including the measures of *E-mails Received* and *E-mails Sent*, respectively. However, our results show that the volume variables are not significantly related to the risk that the employee will develop burnout (p>.1). Similarly, in Model 4, we tested hypotheses H3 and H4 by including the positional variables *Degree* and *Constraint*. Results show that *Degree* is a negative and marginally significant predictor of the burnout index (ß = -.22, p< .05), while constraint is not (p>.1). In Models 5 and 6, we included the behavioral variable hypothesized to be related to disengagement (*E-mails Sent During Out*-of-*office Hours*, *Higher Hierarchical Level Reciprocity* and *Ratio Sent /Received*). We found no support for Hypothesis H5; thus, the ratio of e-mails sent to e-mails received is not related to the risk that employees will develop burnout (p>.1). Consistent with previous results, and confirming Hypothesis H6b (and contrary hypothesis H6a), the more e-mails employees send during out-of-office hours, the lower the risk that they will develop of burnout (ß = -.46 p< .01). Finally, consistent with Hypothesis H7, the higher the number of strong reciprocal ties employees have with other employees from higher hierarchical levels the greater the risk that employees develop burnout (ß = .36 p< .05).

**Table 5:** Linear stepwise regression models–predicting the burnout index (n = 57).

	Model 1	Model 2	Model 3	Model 4	Model 5	Model 6	Model 7	Model 8
Variables	ß (S.E.)	ß (S.E.)	ß (S.E.)	ß (S.E.)	ß (S.E.)	ß (S.E.)	ß (S.E.)	ß (S.E.)
Constant	3.27*** (.20)	3.28*** (.19)	3.24*** (.20)	3.17*** (.22)	3.42*** (.19)	3.26*** (.22)	3.38*** (.22)	3.41*** (.22)
**Step 1: Control Variables**								
Age	.29** (.11)	.29** (.11)	.29** (.11)	.27** (.12)	.27** (.11)	.30** (.14)	.21 (.13)	.22* (.12)
Gender	-.17 (.25)	-.14 (.30)	-.04 (.28)	-.01 (.28)	-.38 (.24)	-.16 (.27)	-.24 (.30)	-.18 (.31)
Executive Hierarchical Level	-.91 (.73)	-.85 (.80)	-.87 (.71)	-.88 (.71)	.08 (.59)	-.91 (.25)	-.12 (.66)	-.01 (.69)
Staff Member Hierarchical Level	-.28 (.26)	-.30 (.28)	-.27 (.26)	-.12 (.34)	-.61 (.25)	-.24 (.31)	-.56* (.38)	-.66* (.36)
**Step 2: Volume Variables**								
E-mails Received		-.04 (.17)					.17 (.20)	
E-mails Sent			-.15 (.14)					.03 (.23)
**Step 3: Position Variables**								
Degree				-.22* (.14)			-.29**(.17)	-.25* (.18)
Constraint				-.19 (.14)			-.03 (.13)	-.03 (.14)
**Step 4: Behavior Variables**								
E-mails Sent During Out-of-office Hours					-.46*** (.11)		-.49*** (.17)	-.44** (.18)
Higher Hierarchical Level Reciprocity					.36** (.13)		.37** (.17)	.42** (.17)
Ratio Sent/Received						-.03 (.17)	-.04 (.18)	-.01 (.17)
Ratio Sent/Received Squared						-.03 (.25)	-.03 (.21)	-.02 (.23)
R^2^	.19	.19	.21	.23	.42	.19	.47	.46
Adjusted R^2^	.12	.11	.13	.14	.35	.09	.34	.32
ΔR^2^		.00	.02	.04	.23	.00	.28	.27
F	2.95**	2.33*	2.70**	2.53**	5.96***	1.92*	3.57***	3.43***

The table presents linear regressions models predicting the variance of a burnout index. Model 1 is the base model with controls. Models 2–3 include controls and each of the volume variables (*E-mails Received* and *E-mails Sent*, which are highly correlated). Models 4–6 include control and each predictor in a stepwise procedure. Model 7–8 are the final model including all variables, using *E-mails Received* and *E-mails Sent* as volume variables, respectively.

Standard errors are robust. Two–tailed tests for all variables.

*p < .1

**p < .05

***p < .01

Models 7 and 8 included all variables hypothesized to be related to the burnout index. The final models explain 32 to 34% of the variance of the burnout index, with the model using *E-mails Received* as a volume variable (Model 7) explaining slightly more of the variance. In the final model, using *E-mails Sent* as a volume variable (Model 8), *Age* was marginally significant (ß = .22, p< .01), in contrast to Model 7, in which it was not significant. *Degree* was negative and significant in Model 7 (ß_model7_ = -.29, p< .05) and marginally significant in Model 8 (ß_model8_ = -.25, p< .1). Hence, the more e-mail communication partners an employee has, the lower the risk that the employee will develop burnout. Finally, consistent with all our previous results, *E-mails Sent During out*-of-*office Hours* (ß_model7_ = -.49 p< .01; ß_model8_ = -.44 p< .05) and *Higher Hierarchical Level Reciprocity* (ß _model7_ = .37, ß _model7_ = .42, p< .05) remained significant in the final models.

In sum, our results suggest that contrary to H1 and H2, none of the volume variables (*E-mails Received* and *E-mails Sent*) are significant predictors of our dependent variables. Contrary to Hypothesis H4a, *Degree* seems to be a negative predictor of exhaustion and burnout risk, while *Constraint* is not significantly related to burnout risk, exhaustion or disengagement (H3b and H4b). *E-mails Sent During Out*-of-*office Hours* are a negative and consistent predictor of burnout risk, exhaustion and disengagement (contrary to H6a and in support of H6b). *Higher Hierarchical Level Reciprocity* is a positive and consistent predictor of exhaustion and burnout risk (H7). Finally, the *Ratio Sent/Received* is not significantly related to burnout risk (H5). Table 6 summarizes how these results relate to our hypothesis.

**Table 6:** Summary of results for hypothesis tests.

Hypothesis		Main Result
**H1**	*The higher the volume of e-mails an employee receives*, *the higher his or her levels of exhaustion and the greater the risk that the employee will develop burnout*.	No support
**H2**	*The higher the volume of e-mails an employee sends*, *the lower his or her levels of disengagement and the lower the risk that the employee will develop burnout*.	No support
**H3**	*a*. *The more central the position of an employee in an e-mail communication network*, *the lower his or her levels of disengagement and the lower the risk that the employee will develop job burnout*. *b*. *The less connected the alters of an employee in an e-mail communication network*, *the lower the levels of disengagement and the lower the risk that the employee will develop job burnout*.	No support
**H4**	*a*. *The more central the position of an employee in an e-mail communication network*, *the higher his or her levels of exhaustion and the higher the risk that the employee will develop job burnout*. *b*. *The less connected the alters of an employee in an e-mail communication network*, *the higher the levels of exhaustion and the higher the risk that the employee will develop burnout*.	Contrary to H4a, the more central the employee’s position the lower the levels of exhaustion and burnout. No support for H4b.
**H5**	*The ratio of e-mails sent versus e-mails received will have a curvilinear relation to the risk of burnout experienced by employees*, *such that lower and higher values for the ratio will be associated with a higher risk that the employee will develop burnout*.	No support
**H6**	*a*. *The more an employee sends e-mails during out-of-office hours (nights*, *holidays and weekends*,*) the higher his or her levels of exhaustion and the higher the risk that the employee will develop burnout*. *b*. *The more an employee sends e-mails during out-of-office hours (nights*, *holidays and weekends)*, *the lower his or her levels of disengagement and the lower the risk that the employee will develop burnout*.	In support of H6b and contrary to H6a, the greater the number of e-mails sent during out-of-office hours the lower the levels of exhaustion, disengagement and burnout.
**H7**	*The higher the number of reciprocated e-mail communication ties an employee has with hierarchical superiors*, *the higher his or her level of exhaustion and the higher the risk that the employee will develop burnout*.	Full Support

### Logistic regressions–identifying employees with higher risk of burnout

In response to calls to identify employees with higher levels of exhaustion, disengagement or burnout (see: [11]), we estimated penalized logistic regressions models to distinguish employees with a higher risk of experiencing burnout from their lower-risk colleagues. Table 7 presents the results of our predictions. For each dependent variable, we dichotomized the values of exhaustion, disengagement and burnout index at the mean plus one standard deviation. Because there are fewer employees with higher levels of burnout or its dimensions, than employees with lower levels, our sample is skewed. As such, we report the recall precision and F1 statistics for all our models. Note that our goal was to maximize the recall of the model, in order to make sure that as many employees with higher levels of burnout are identified, while retaining a reasonable level of precision.

**Table 7:** Penalized logistic regression prediction results for burnout, exhaustion and disengagement (n = 57).

	Burnout	Exhaustion	Disengagement
**True Positive**	8	8	7
**True Negative**	46	45	37
**False Positive**	3	3	11
**False Negative**	0	1	2
**Recall**	100%	89%	78%
**Precision**	73%	73%	39%
**F1 Score**	84%	80%	52%

AUC statistics are .96 for burnout, .96 for exhaustion and .84 for disengagement. The Hosmer-Lemeshow GOF test is non-significant for all models. The prediction threshold is .3 for burnout and for disengagement and .4 for exhaustion.

For exhaustion, the model correctly identifies eight out of nine employees with higher levels of exhaustion, and misclassifies three out of 48 employees with lower levels of exhaustion (recall 89% and precision of 73%). For disengagement, the model correctly identifies seven out of nine employees with higher levels of disengagement and misclassifies eleven out of 48 employees with lower level of disengagement (recall of 78% but precision of 39%).

To assess the accuracy of our logistic regression models we tested each for discrimination and calibration [63–65]. We computed the AUC statistic [63, 66–67] in order to evaluate discrimination; that is, how good our models were at separately identifying individuals experiencing exhaustion, disengagement or a high risk of developing burnout, from those not experiencing them. Results for the AUC statistic suggest that all fitted models for exhaustion and burnout were excellent at discriminating between at–risk and not-at-risk individuals (Exhaustion = .96; Burnout = .96). The model for disengagement has an acceptable value for the AUC statistic (i.e., .84).

To evaluate calibration–or the extent to which our predicted probabilities coincided with actual observed prevalence in our sample–we used the Hosmer-Lemeshow statistic [63–65]. The results suggest that all fitted models were well calibrated. Test values for all fitted models were greater than a significance level of .05, meaning that there is insufficient evidence to conclude that the models do not accurately fit the data (Exhaustion = .8; Disengagement = .7; Burnout = .37).

### Additional analyses

We undertook several additional analyses (available from the authors) to assess the robustness of our results. First, we initially removed one employee from our analyses who had sent an extremely large number of e-mails during the period of analysis. We subsequently estimated all linear regression and logistic regression models again, this time including this employee in the data. Our results were substantively unchanged when this outlier was included. Second, we recomputed exhaustion, disengagement and the burnout index using only the items with factor loadings higher than 0.7. Results using two items for exhaustion (items 10 and 12) and four items for disengagement (items 1, 6, 9 and 11) were substantively identical to those reported here. Third, we calculated three different normalized values of *E-mails Sent During Out-of-office Hours*, using as denominators the total number of e-mails sent, the daily average number of e-mails sent, and finally, the daily average number of communication exchanges (e-mails sent plus e-mails received). In all cases, results were consistent with those presented earlier. Fourth, we estimated all models again using three–month and one–month observation periods for the e-mail data. In general, our results proved to be robust and consistent under these different time frames. Even though the indicator *E-mails Sent During Out*-of-*office Hours* lost significance in the one–month linear regression models, this might have been the result of insufficient data being available to accurately assess the indicator for the shorter period.

We further tested the potential impact of time on our results, specifically the possibility that more recent exchanges might reveal more about the risk of experiencing burnout. In order to time–weight our data, we used the approach developed by Lerner, Baussman, Snijders and Brandes [68], with a 90-day half-life parameter, and an amplification factor ranging from 1 to 2 in .25 steps between January and May. Results demonstrated that the significance of linear models R^2^ and the significance of the relevant variables fell, remaining, however, consistent with previous findings. Logistic regressions lost some predictive capacity but the overall success of all models remained high. Overall, these additional analyses regarding the temporal dimension of the relationship between e-mail communication patterns and burnout suggest that burnout is better captured by regular, rather than more recent communication patterns.

Finally, the popular press suggests that mass e-mail communications received by employees, and excessive use of the cc. function can be costly for the individual. Since all e-mails need to be read, spending time on communications that are not directed to the recipient can be considered an additional demand. As such, receiving many mass communications that have little relevance for individual employees increases the demands they feel [69]. This might happen mainly because they have to search, read, sort and manage or delete them while also keeping track of the smaller number of e-mails that might occasionally require some action. It is therefore possible that receiving a large number of mass–communications and being cc.ed into many messages might affect employee risk of burnout. To assess the extent to which this possibility holds in our data, we created a general indicator of the number of mass e-mail communications and cc.s received by each employee, by counting the number of times an employee was a recipient of an e-mail sent to more than four individuals. We then used this variable as a single predictor of the burnout index and of each of its constituent dimensions, and as an additional variable in our previous regressions. Results consistently suggest that in our data receiving mass e-mail communications is not predictive of a high risk of developing burnout.

## Discussion

The purpose of this paper is to propose a way to identify employees at risk of experiencing burnout by using their e-mail communications. Building on the extant literature and using the JD-R model as our conceptual framework, we propose that the risk of burnout may be related to the volume of e-mail communications that employees deal with, to their position in an e-mail communication network, or to specific communication behavior related to e-mails. Our results contribute to the overall aim of the research, though they offer only partial support for the hypothesized relationships between burnout and our study variables.

Linear regressions proved capable of explaining 37%, 19% and 34% of the variance of exhaustion, disengagement and of the burnout index, respectively. Furthermore, logistic regressions show that our models were able to identify employees with higher levels of exhaustion, disengagement and higher risk of burnout and distinguish them from employees with lower risk, with a high level of recall (89%, 78% and 100% respectively). The small sample size, the observation window, and the relatively coarse indicators of e-mail communication mean that these results are quite encouraging, given our attempt to provide a simple and cost-effective way of identifying individuals at risk of developing burnout in organizations.

Our results indicate that volume–related e-mail communication variables are poor predictors of increased risk of burnout and levels of exhaustion or disengagement. This is consistent with previous research suggesting that it is the employee’s perception of the volume of e-mail communication rather than its actual volume that relates to employee stress or burnout [43, 44]. Positional variables, especially degree, are slightly better predictors, but the relationship with exhaustion or burnout is the opposite of what we predicted. That is, a higher degree is related to lower levels of exhaustion or risk of burnout, suggesting that, at least in this case, the number of e-mail exchange partners is not a good representation of the job demands that organizations place on employees and might alternatively be a representation of social support; in other words, a job resource (see: [4]). If this is the case, then higher numbers of communications partners might in fact buffer the impact of job demands on employees.

Behavioral variables were good predictors of exhaustion, disengagement and risk of burnout, especially the number of e-mails sent during out-of-office hours and the number of reciprocal strong ties with higher hierarchical levels. However, the number of e-mails sent during out-of-office hours, contrary to what we predicted, is negatively related to exhaustion, disengagement and risk of burnout. Why this is the case is not clear and we have no information that allows us to provide a definitive explanation. However, we propose two different explanations for this result. First, it is possible to interpret the results as showing that employees with higher levels of job burnout may reduce the amount of e-mails they send during out-of-office hours. This suggests that refraining from sending e-mails at such times is a consequence of job burnout. According to this view, an employee´s decision to cope with strain by reducing the amount of e-mails they send would explain our counterintuitive result. This would be consistent with previous research suggesting that individuals retreat from work as a strategy to protect themselves (see: [4]). An alternative explanation of this result might be that employees using their free time to write e-mails are, as suggested by Barley et al. [42], making use of a compensatory strategy to control their job demands. Additionally, employees may find e-mails to be a good tool for controlling and pre-organizing the work they will be dealing with during their normal working hours, thus buffering job demands. If this second interpretation is correct, it may suggest that attempts to restrict employee discretion at managing e-mails during out-of-office hours may, in fact, reduce their autonomy and control over their workload. This would be consistent with previous work suggesting that not sending e-mails during out-of-office hours may relate to increased burnout levels [70]. Additional research is needed to provide a more definitive interpretation of the result.

Finally, the number of strong reciprocal ties with higher hierarchical levels is positively related to exhaustion and to the risk of developing burnout, suggesting that hierarchical e-mail communications relate to increased levels of demand on employees. While earlier literature argued that supervisor feedback provides social support to employees (see: [11]), our results suggest that frequent and reciprocal e-mail communication with superiors captures the demands placed on employees and the control exercised by supervisors. As Derks and Bakker [23] suggest, social support via feedback is more likely to be delivered by way of a more personal means of communication, which would exclude e-mail. Moreover, if e-mail is used to provide direct, harsh and demanding negative feedback to subordinates [23], then frequent and reciprocal e-mail communications with supervisors would become a potentially more demanding and emotionally challenging task.

Overall, the non–significant results for volume variables and the significant results for communication behavior variables in the linear regressions support previous work in suggesting that e-mail-use is not necessarily by itself a cause of burnout but that, on the contrary, e-mails can function either as a job resource or a job demand [23]. In line with previous findings, the effect of the numbers of e-mails on stress and burnout might be mediated by employee perceptions of volume [44], overload [43, 45], the usefulness of technology [42], and work spillover [28]. While the relationship between reported communication during out-of-office hours and strain has been reported in earlier literature [28, 42, 56], those studies were based on employee perceptions of their communications or short spells of communication exchanges. In this study, our direct method of collecting the rate of exchanges which take place out-of-office hours provides a more accurate and reliable measure of individuals’ work outside normal established hours and, thus, provides a more systematic means of relating this to the risk of developing burnout.

### Limitations

Despite the encouraging nature of our results, this study has some limitations. First, there were no individuals with extreme levels of burnout included in our dataset, thus preventing us from assessing whether the model would be able to identify employees who are experiencing the syndrome. While this is a (fortunate) limitation, we believe that it suits the purpose of this paper to identify employees *at risk* of developing burnout. The identification of employees with high, but not extreme, values of exhaustion and disengagement implies that it is still possible for these employees to receive help and support from the organization in order to prevent them experiencing the syndrome.

Second, while our models are relatively good at predicting exhaustion and overall risk of burnout, they do not predict disengagement well. This might be because exhaustion is the most salient dimension of burnout [18], because e-mails are more likely to be related to demands than resources, or because e-mails are inherently poor predictors of disengagement. It is also possible that our indicators were too crude, and that more refined measurements are required to improve predictions. However, we argue that predicting exhaustion is more critical to identifying employees at risk of developing burnout than to predicting disengagement. In their conceptual model, Leiter & Maslach [71] propose that exhaustion could be a precursor of disengagement and that burnout emerges when both exhaustion and disengagement are present. As such, the ability to identify employees with higher levels of exhaustion provides a better earlier warning signal than identifying employees with disengagement.

Third, another limitation of the study comes from the data. This study was conducted in only one company and with a limited number of employees. The fact that all data were collected from the same company prevents the statistical generalization of our results. Our sample size is also clearly a limitation. A larger sample size would reduce the influence of idiosyncrasies in a small dataset and offer a more compelling validation of our indicators, and it would provide a definitive interpretation of our control variables (especially age and gender). Additionally, a larger sample would have allowed us to obtain robust results, avoiding potential overfitting problems in our logistic regression models, which we mitigated using penalized logistic regression. However, an ability to explain such a high percentage of variance and to identify individuals with greater risk of burnout in such a small sample, and with high levels of recall, actually constitutes a conservative test of our predictors. In this sense, larger sample sizes could mean that additional variables reach significance and therefore provide enhanced predictions.

### Directions for future research

Further research is required to verify whether our results can be replicated in different contexts. We would encourage researchers to collect e-mail communication and burnout data from larger groups of employees in multiple companies to replicate our research. In these additional data-collection efforts, we would encourage researchers to adopt a longitudinal design in the collection of burnout related data, covering the same period as the e-mail communication data. This would enable the development of more precise indicators and predictions in three ways: (i) by investigating whether e-mail communication patterns (or changes in patterns) can identify changes in burnout levels over time (e.g., delta e-mail is related to delta burnout), (ii) by exploring whether more sophisticated indicators of e-mail communications might act as even more precise predictors of burnout when the evolution of the patterns is considered over time, and (iii) by possibly determining whether a causal relationship exists between burnout and e-mail communication patterns (and if so, its direction).

Finally, researchers could attempt to obtain the contents of the e-mail communications in order to derive better predictions of employee burnout from e-mail data. Information about the type of messages employees send/receive might allow researchers to more accurately classify e-mails as either resources or demands. Moreover, it would help them to establish whether the other side of information overload (i.e., qualitative overload) [23,24, 29,54] might affect employees and whether, as proposed by Derks and Bakker [23], there is any specific relation between e-mail content and levels of job burnout.

### Conclusion

This paper proposes an innovative way of identifying employees at risk of experiencing burnout in an organizational setting. While burnout is prevalent in organizations and has severe negative consequences for employees and organizations, it is difficult to detect and, therefore, prevent. Using e-mail communication and burnout data collected through an online survey, we develop models that are capable of explaining a considerable amount of the variance of employee burnout and exhaustion and of identifying, with high recall, which employees are at risk of exhaustion and burnout. We believe that our results provide strong support that can help us achieve the main goal of the study, namely, to propose a cost-effective early warning system for burnout. Monitoring employee e-mails appears to be an unobtrusive and relatively effective way of identifying employees at risk. Clearly, this first step in the identification of burnout in employees should be complemented by additional steps, such as interviews or the administration of survey questionnaires in order to confirm the emergence of the syndrome. It is not realistic to believe that monitoring e-mail communication patterns could be completely reliable, but even if it were only used as a first step in a prevention strategy, it would be worthwhile to develop such an early warning system.
